# Chemical Constituents of *Excoecaria acerifolia* and Their Bioactivities

**DOI:** 10.3390/molecules15042178

**Published:** 2010-03-26

**Authors:** Yan-Li Zhao, Qiu-Xia He, Yang Li, Si-Feng Wang, Ke-Chun Liu, Yong-Ping Yang, Xiao-Li Li

**Affiliations:** 1 Laboratory of Ethnobotany, Kunming Institute of Botany, Chinese Academy of Sciences, Kunming 650204, Yunnan, China; E-Mails: zhaoyanli@mail.kib.ac.cn (Y.L.Z.); liyang@mail.kib.ac.cn (Y.L.);; 2 Graduate University of Chinese Academy of Sciences, Beijing 100039, China;; 3 Biology Institute of Shandong Academy of Sciences, Jinan 250014, Shandong, China; E-Mails: heqiuxia8008@163.com (Q.X.H.); peakwang10@gmail.com (S.F.W.); hliukch@keylab.net (K.C.L.); 4 Institute of Tibetan Plateau Research at Kunming, Kunming Institute of Botany, Chinese Academy of Sciences, Kunming 650204, Yunnan, China

**Keywords:** kaurane diterpenoid, *Excoecaria acerifolia*, NMR, antiangiogenic, antiproliferative

## Abstract

A new kaurane diterpenoid, 3α,18-dihydroxy-3β,20-epoxykaur-15-ene (**1**), was isolated from the aerial parts of *Excoecaria acerifolia* (Euphorbiaceae) together with 16 known compounds. Their structures were identified by extensive spectral analysis, especially 2D NMR techniques. Antiangiogenic effects of compounds **1-6** and **9-17** were evaluated using a zebrafish model, with compound **9** being active in this bioassay. At the same time, compounds **4**, **6**, **10**, **11** showed activity in inhibiting the growth of A549 lung cancer cells, and the compound **10** also showed apoptosis-inducing effects on A549 lung cancer cells.

## 1. Introduction

**Figure 1:**
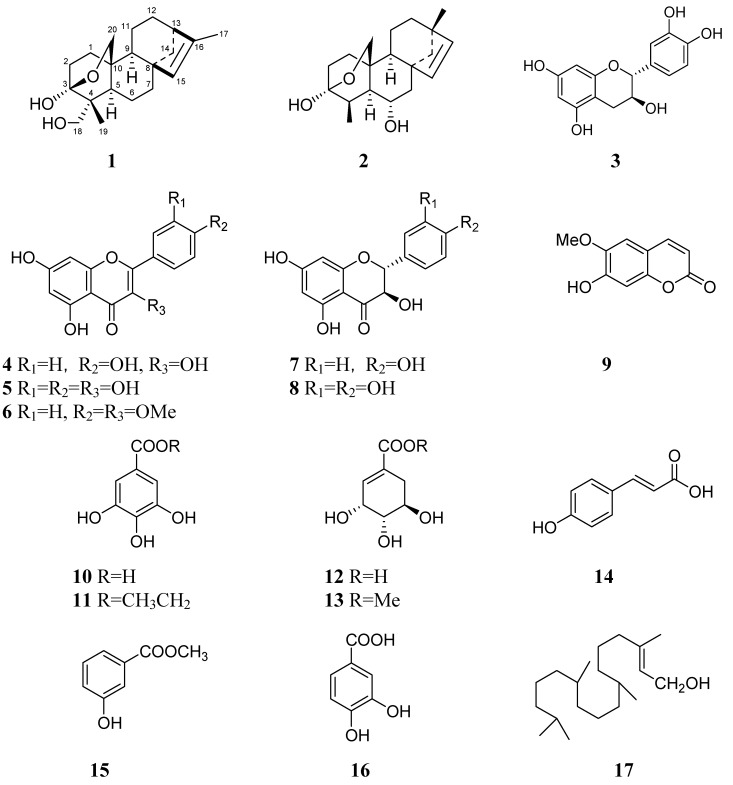
The structures of compounds **1-17**.

The genus *Excoecaria* of the plant family Euphorbiaceae, comprising 40 species, is wildly distributed in tropical Asia, Africa and Oceania. In China, there are five recorded *Excoecaria* species, of which *E. agallocha *has been well studied and a number of novel diterpenoids with anti-tumor bioactivity [1] and anti-HIV phorbol ester [2] found in it have been reported. However, no information concerning the chemical constituents of *E. acerifolia* has been published so far. The *E. acerifolia* plant is a dominant species of Maquis vegetation type distributed in the dry valleys in Southwest China [3,4]. Aiming to search for bioactive secondary products from *E. acerifolia*, we investigated the aerial parts of this plant and this has led to the isolation of a new compound, 3α,18-dihydroxy-3β,20-epoxykaur-15-ene (**1**), and 16 known compounds, including 3β,20-epoxy-3α,6α-dihydroxy-18-nor-beyer-15-ene (**2**) [5], catechin (**3**) [6], kaempferol (**4**) [7], quercetin (**5**) [8], 5,7-dihydroxy-3,4'-dimethoxyflavone (**6**) [9], aromadendrin (**7**) [10], texifolin (**8**) [11], 6-dimethoxy-7-dihydroxycoumarin (**9**) [12], trihydroxybenzoic acid (**10**) [13], progallin A (**11**) [14], shikimic acid (**12**) [15], shikimic acid Me ester (**13**) [15], (*E*)-*p*-coumatic acid (**14**) [16], *m*-hydroxybenzoic acid Me ester (**15**) [17], 3,4-dihydroxy-benzoic acid (**16**) [18] and phytol (**17**) [19] (Figure 1). This paper presented the results in the separation and structure elucidation of the new kaurane diterpenoid, and the antiangiogenic activities using a zebrafish model and the antiproliferative activities using A549 lung cancer cells of compounds **1-6** and **9-17**.

## 2. Results and Discussion

Compound **1** was isolated as white crystals from methanol. Its HRESIMS indicated the molecular formula C_20_H_30_O_3_, as evidenced by the pseudo-molecular ion peak at m/z 341.2099 [M+Na]^+ ^(calcd. 341.2092), corresponding to six degrees of unsaturation. IR absorption bands at 3,381, 2,919, 1,646 and 1,058 cm^−1^ implied the presence of hydroxyl, methylene, olefinic and ether groups, respectively. The ^1^H-NMR spectra exhibited two methyl groups as singlets (δ_H_1.68, 1.15). The ^13^C-NMR and DEPT spectra of **1** in CDCl_3_ showed 20 carbons with 28 directly attached protons for the diterpene nucleus (Table 1). The ^1^H- and ^13^C-NMR spectra also showed the presence of a double bond, four tertiary carbons, and three oxygenated carbons in this molecule. Further analysis of 2D NMR spectra (^1^H-^1^H COSY, HMQC, ROESY and HMBC) lead to the construction of a structural formula for **1** based on an anthracene skeleton with an epoxy ring formed between C-3 and C-20, *cis*-olefins at C-8 and C-13, and a hydroxyl methyl groups at C-18 to complete a kaurane skeleton, which was also supported by the characteristic peak of two angular methyl groups at δ_H_1.68 (3H, s) and 1.15 (3H, s) [20,21]. The comparison of the 1D NMR data of **1** with those of the known compound excoecarin D [22], which was determined by X-ray diffraction, showed the ^1^H- and ^13^C-NMR data of these two compounds were structurally similar. The differences can be rationalized to the position change of methyl-17 from C-13 in excoecarin D to C-16 in **1**, which lead to the observation of only one olefinic proton (δ_H_ 5.04) in **1** and one methine at C-13 in **1** other than a quaternary carbon in excoecarin D. This deduction was established by the HMBC correlations of the methyl group (δ_H _1.68, C-17) with δ_C _42.1 (C-13), 133.3 (C-15) and 142.4 (C-16). 

The relative configurations of **1** were determined on the basis of ROESY experiments. The olefinic proton at C-15 correlated with the methylene protons at C-20 and the protons at CH_3_-17. ROESY correlations of H-5 with the protons of the oxygenated methylene (C-18) and H_2_-20 with H-19 indicated C-18 were α-orientated. ROESY correlations of H-9 with H_2_-14 showed both H-9 and C-14 are on the same face of the molecule with α-orientation. Therefore, the structure of **1** was established as 3α, 18-dihydroxy-3β,20-epoxykaur-15-ene.

**Table 1:** The NMR data of **1** (in CDCl_3_).

Position	δ_C_	δ_H_	Position	δ_C_	δ_H_
1a	30.2 (t)	1.18 (m)	11a	19.2 (t)	1.12 (m)
1b		1.58 (m)	11b		1.49 (m)
2a	24.5 (t)	1.61 (m)	12a	34.9 (t)	1.18 (m)
2b		1.40 (m)	12b		1.58 (m)
3	98.6 (s)	/	13	42.1 (d)	2.19 (d, 5.7)
4	43.9 (s)	/	14a	44.7 (t)	1.12 (m)
5	45.9 (d)	1.43(m)	14b		1.49 (m)
6a	20.6 (t)	1.12 (m)	15	133.3 (d)	5.04 (s)
6b		1.49 (m)	16	142.4 (s)	/
7a	36.4 (t)	1.78 (m)	17	15.4 (q)	1.68 (s)
7b		1.53 (m)	18a	70.2 (t)	3.66 (d, 11.0)
8	48.2 (s)	/	18b		3.30 (d, 11.0)
9	43.8 (d)	1.46 (m)	19	13.4 (q)	1.15 (s)
10	36.5 (s)	/	20a	68.0 (t)	4.43 (dd, 8.5, 2.0)
			20b		3.86 (dd, 8.5, 2.0)

^a^ Spectra were obtained at 400 (^1^H-NMR) and 100 (^13^C-NMR) MHz in CDCl_3_ and chemical shifts (δ) are in ppm with *J* values in Hz.

**Figure 2:**
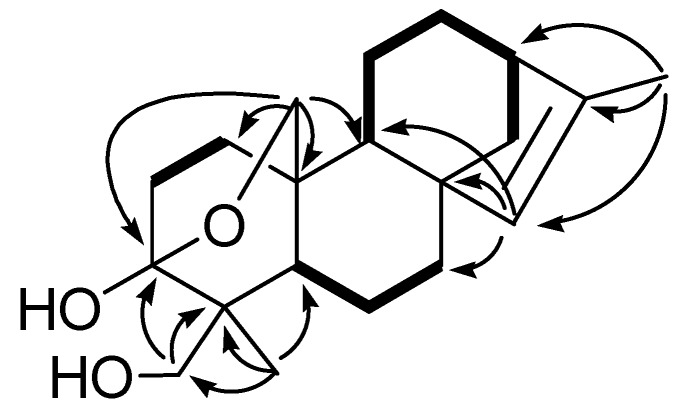
Key HMBC (→) and ^1^H, ^1^H COSY (—) correlations of **1**.

### Biological Acticity

**Figure 3:**
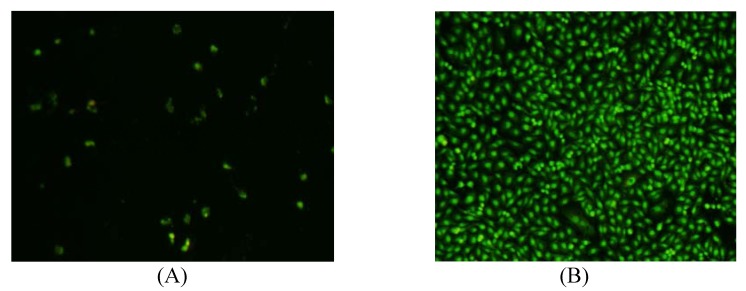
Photos of acridine orange staining (A: compound **10**, B: control).

The antiangiogenic activities of compounds **1-6** and **9-17** were evaluated using a zebrafish model, in terms of the inhibition on the growth of intersegmental vessels, with PTK787 as positive control (IC_50_0.15 μg/mL) [23]. The results showed that intersegmental vessels of embryos treated with compound **9** was significantly less than that of the control (0.1% DMSO in sterile salt water). The inhibition ratio of compound **9 **was 64.9% at a concentration of 50 μg/mL (Table 2). The antiproliferative activities of compounds **1-6** and **9-17 **were evaluated using A549 lung cancer cells by MTT assay [24]. The results indicated that compounds **4**, **6**, **10** and **11 **showed a certain extent antiproliferative activities (Table 3). From the photos of acridine orange staining, compound **10** showed obvious effect of inducing apoptosis of A549 lung cancer cells (Figure 3). Other tested compounds didn’t show any obvious antiangiogenic and antiproliferative bioactivities.

**Table 2:** Anti-angiogenic activity of compounds **1**-**6**, **9**-**17.**

Compound	Concentration (μg/mL)	Intersegmental Vessels (ISV)	Inhibition Ratio (%)
**1**	100	24.7 ± 1.2	8.0
**2**	100	26.7 ± 1.2	0.5
**3**	100	26.0 ± 0.0	3.0
**4**	100	24.7 ± 1.2	8.0
**5**	100	24.7 ± 1.2	8.0
**6**	100	20.0 ± 2.8	25.4
**9**	50	9.4 ± 8.4 **	64.9
**10**	100	26.7 ± 1.2	0.5
**11**	100	26.7 ± 1.2	0.5
**12**	100	26.3 ± 1.5	1.7
**13**	100	24.7 ± 0.6	8.0
**14**	100	26.3 ± 0.6	1.7
**15**	100	25.0 ± 0.0	6.7
**16**	100	24.7 ± 2.3	8.0
**17**	100	27.0 ± 1.0	0
control *^a^*		26.8 ± 1.8	
PTK787	10	0 **	100

*^a ^*0.1% DMSO in sterile salt water; ** means that the value was significantly different from the control and *p *< 0.01.

**Table 3:** Inhibitory effect of compounds **4, 6, 10, 11** on the proliferation of A549 lung cancer cells.

M(%) ^a^/ Compounds	Concentration		
	25 μg/mL	50 μg/mL	100 μg/mL
**4**	12.78 ± 6.56	40.8 ± 2.96**	52.51 ± 2.97**
**6**	14.35 ± 0.61**	48.76 ± 5.08**	62.03 ± 5.72**
**10**	15.92 ± 3.12**	61.13 ± 2.49**	74.6 ± 2.28**
**11**	22.38 ± 8.37	26.47 ± 5.25**	48.6 ± 6.76**

^a ^M (%) means the mean value of inhibition ratio, *, p < 0.05, **, p < 0.01, compared with control group.

## 3. Experimental

### 3.1. General

1D and 2D NMR experiments were performed on a Bruker AM-400 or DRX-500 spectrometer. Chemical shifts (δ) are expressed in ppm with reference to the solvent signals. Mass spectra were recorded on a VG Autospec-300 spectrometer under 70 eV. Optical rotation was measured with a Horiba SEPA-300 polarimeter. A Bio-Rad FTS-135 spectrophotometer was used for scanning IR spectroscopy of compounds with KBr pellets. Column chromatography was performed on silica gel (200-300 mesh, Qingdao Marine Chemical Inc., Qingdao, People’s Republic of China), silica gel H (10–40 μm, Qingdao Marine Chemical Inc.) and MCI gel CHP20P (75–150 μm, Mitsubishi Chemical Corporation, Tokyo, Japan). Fractions were monitored by TLC and spots were visualized by heating plates spraying with 15% H_2_SO_4_ in EtOH.

### 3.2. Plant Material

The aerial parts of *E. acerifolia *were collected from Deqin, Yunnan Province, People’s Republic of China. The plant material was identified by Dr. Yuanwen Duan. A voucher specimen (No.Yangyp 080724) was deposited at Kunming Institute of Botany, Chinese Academy of Sciences.

### 3.3. Extraction and Isolation

**The dry aerial parts of *E. acerifolia *(35 kg) were powdered and extracted with 70% aqueous acetone (3 × 25 L) for 24 h at room temperature. The solvent was concentrated *in vacuo* to give a crude extract. The extract was then dissolved in H_2_O and partitioned with EtOAc. The EtOAc portion was subjected to column chromatography over MCI gel eluting with 95% EtOH and concentrated *in vacuo*. The residue (1532g) was the subjected to column chromatography over silica gel (80−100 mesh), eluting with petroleum ether−Me_2_CO (from 1:0 to 1:1) to afford fractions A−D. Fraction A was subjected to column chromatography over silica gel (petroleum ether/acetone) to yield **17 **(385 mg). Fraction B was subjected to column chromatography over silica gel (CHCl_3_/Me_2_CO) and Sephadex LH-20 (CHCl_3_/MeOH) to give **1** (27 mg), **2** (9 mg), **4 **(1,369 mg),** 5** (565 mg),** 9 **(7 mg), **10** (945 mg),** 11** (187 mg),** 12** (143 mg), **14** (12 mg),** 15** (40 mg). Fraction C was subjected to column chromatography over silica gel (CHCl_3_/Me_2_CO) and Sephadex LH-20 (CHCl_3_/MeOH) to obtain **3** (21 mg), **6** (4 mg),** 7** (3 mg),** 8** (2 mg), **13** (73 mg). Fraction D was purified by repeated column chromatography over silica gel (CHCl_3_/MeOH) to afford **16 **(46 mg).

*Compound* **1**: white crystals; mp 189.2-190.7; [α]^25.1^_D_ = -34.5 (*c* 0.85, CHCl_3_); UV_max_ (CHCl_3_): 198, 205, 240 nm; IR (KBr);*ν*_max_ 3381, 2919, 1461, 1444, 1318, 1194, 1123, 1058, 1040 cm^−1^; For ^1^H- and ^13^C-NMR see Table 1; HRESIMS *m*/*z *341.2099 (calcd for C_20_H_30_O_3_Na, 341.2092).

### 3.4. Antiangiogenesis and antiproliferative assays

#### 3.4.1. Antiangiogenesis [23]

Stock solutions (10 mg/mL) of all samples were prepared by dissolving the test compounds in 100% DMSO. These solutions were diluted in sterile salt water (5 mM NaCl, 0.17 mM KCl, 0.4 mM CaCl_2_, 0.16 mM MgSO_4_) to obtain solutions with the test compounds dissolved in 0.1% DMSO. These solutions were aliquoted into 96-well plates, and embryos at 24 hpf (hours post fertilization) were also transferred randomly into the above wells. After 24h of treatment, the intersegmental vessels of embryos were visualized with green fluorescent protein labeling and endogenous alkaline phosphatase staining. The antiangiogenic activities of compounds were calculated from the inhibition ratio of angiogenesis. PTK787 was used as the positive control. 

#### 3.4.2. Antiproliferative [24]

A549 lung cancer cells were cultured in RPMI 1640 medium at 37 °C with 5% CO_2 _and 95% air, supplemented with 10% (v/v) bovine calf serum and 80U/ml penicillin/streptomycin. The cells were seeded onto 96-well plates and treated with compounds at 25, 50 and 100μg/mL for 48h, respectively. Cell viability was determined by MTT (3-(4,5-dimethylthiazol-2-yl)-2,5,-diphenyltetrazolium) assay according to Price et al. The light absorption was measured at 570nm using Spectra MAX 190 microplate spectrophotometer (GMI Co., USA). Inhibition rate was calculated by the formula: 

Inhibition (%) = 100% -(OD _treatment_ - OD _blank_)/(OD _control_- OD _blank_) ×100%

The cells were incubated with compounds (100 μg/mL) for 48 h, and stained with 0.1mg/mL of acridine orange (AO) at room temperature for 5 min. Then the cells were observed and photographed using the fluorescent stereo microscope (Olympus, Japan).

## 4. Conclusions

Our current research led to the isolation of a new kaurane diterpenoid (**1**), together with 16 known compounds **2**-**17** from the aerial parts of *Excoecaria acerifolia*. Their structures were identified by extensive spectral evidence, including HSQC, HMBC, ^1^H-^1^H COSY, ROESY experiments. Most of isolated constituents are phenolic compounds, which hints that the major chemical constituents in the aerial parts of *Excoecaria acerifolia* may be phenolics. The antiangiogenic and antiproliferative activities of part of isolates were evaluated and some of them are bioactive.
